# The impact of gender on emotional reactions, perceived susceptibility and perceived knowledge about COVID-19 among the Israeli public

**DOI:** 10.1093/inthealth/ihaa101

**Published:** 2021-01-15

**Authors:** Inbar Levkovich, Shiri Shinan-Altman

**Affiliations:** Oranim Academic College of Education, Israel; The Louis and Gabi Weisfeld School of Social Work, Bar Ilan University, Israel

**Keywords:** COVID-19, emotional reactions, gender differences, perceived knowledge about COVID-19 risk factors, perceived susceptibility, precautionary behaviour

## Abstract

**Background:**

The current COVID-19 outbreak is seriously affecting the lives and health of people across the globe. While gender remains a key determinant of health, attempts to address the gendered dimensions of health face complex challenges.

**Methods:**

In a cross-sectional study 482 participants (men=237, women=245) completed questionnaires on precautionary behaviour, perceived knowledge about COVID-19 risk factors, emotional reactions toward COVID-19 and perceived susceptibility. We examined gender differences in perceived knowledge about COVID-19 risk factors, healthy behaviours, threat perceptions and emotional responses, as well as the role of gender as a moderating factor.

**Results:**

Women reported higher levels of precautionary behaviour (*t*(475)=3.91, p<0.001) and more negative emotional reactions toward COVID-19 (*t*(475)=6.07, p<0.001). No gender differences emerged in perceived susceptibility or knowledge about COVID-19. The multiple regression model is significant and explains 30% of the variance in precautionary behaviour, which was found to be higher among women and older participants, those with higher perceived knowledge about COVID-19 risk factors and those with higher emotional reactions. Gender exhibited a significant moderating role in the relationship between perceived knowledge and precautionary behaviour (B=0.16, SE=0.07, β=0.13, p=0.02, 95% CI 0.03 to 0.30).

**Conclusion:**

Women exhibited higher levels of precautionary behaviour and emotional responses.

## Introduction

The novel coronavirus, 2019-nCoV, first emerged as a human pathogen in the city of Wuhan in China's Hubei province.^1–3^ Compared with severe acute respiratory syndrome (SARS)-CoV, 2019-nCoV appears to be more readily transmitted from human to human. On 20 January 2020, after the disease had spread to multiple countries, the WHO declared a Public Health Emergency of International Concern (PHEIC),^4^ the sixth PHEIC to be declared under International Health Regulations.

The first Israeli was diagnosed with COVID-19 in February 2020. Since then, 200 000 Israelis have been diagnosed with the virus and >1000 people have died. The Israel Ministry of Health began releasing guidelines determining a new daily routine for the public. Like other countries, since the onset of the epidemic, Israel has implemented diverse containment measures, including quarantine.^5,6^

Recognising the extent to which a disease outbreak differentially affects women and men is fundamental for understanding the primary and secondary effects of a health emergency on different individuals and communities and for creating effective and equitable policies and interventions. Although gender-disaggregated data for COVID-19 so far show equal numbers of cases for men and women, there seem to be gender differences in mortality and vulnerability to the disease.^7^ For example, the death rate in China was 2.8% in men compared with 1.7% in women (n=44 000 people).^2,8^ Emerging evidence suggests that more men than women are dying, conceivably due to gender-based immunological or gendered differences such as smoking patterns and prevalence.^9,10^

Research on COVID-19 is still in its infancy. Empirical evidence reveals gender differences in the general population regarding perceived knowledge about COVID-19 risk factors, responses to prevention guidelines and emotional responses to the situation. A study from China conducted during the COVID-19 pandemic showed that women were better informed about the disease than men and complied more with official guidelines, such as wearing masks and avoiding public spaces.^11^ In a study that investigated behavioural patterns and attitudes related to SARS prevention among 839 Hong Kong adults,^12^ up to 67.3% of male and 78.8% of female respondents reported wearing masks in public places in Hong Kong all or most of the time during the 2 wk preceding the study. Eight studies that reviewed evidence of handwashing compliance in community settings during the SARS outbreak found significant gender differences in handwashing compliance, with women being more compliant than men.^13^

Individuals who feel especially vulnerable to infectious diseases often exaggerate their responses in the form of disgust, negative perceptions and avoidance of those they perceive as a threat to their health.^14,15^ Comparisons between men and women reveal differences in the prevalence of several infectious diseases and different immunological responses. One study showed that perceived infectability decreases up to the age of 50 y then increases among women from that age onward. Gender differences are only significant in younger participants, with women scoring higher on perceived vulnerability than men.^16^ Women's greater perceived vulnerability to disease may be based on the history of developmental psychology and on current exposure to disease threat.^17^

Female gender has been identified as the most potent predictor of anxiety, fear and stress during the current pandemic,^18^ with levels of anxiety disorder three times higher among women than among men.^19^ This gender bias is supported by evidence of gender differences in stress response systems. Moreover, women also appear to be more susceptible to social isolation.^20^ Among the gender-related factors are the predominant roles played by women as family caregivers and as frontline healthcare workers.^21^ For example, Thelwall and Thelwall^22^ analysed tweets about COVID-19 during 10–23 March 2020. The results show that women are more likely to tweet about the virus in the context of family, social distancing and healthcare, whereas men are more likely to tweet about sports cancellations, the global spread of the virus and political reactions. During the SARS outbreak, more women than men sought out psychological counselling and these consultations focused mainly on emotional issues.^23^

Overall, research suggests that while COVID-19 infects almost an equal number of men and women, women appear to be less likely to die from the virus than men. Nevertheless, studies on previous epidemics show that the psychological responses of women are more severe.

The objectives of the current study are: (1) to examine gender differences in perceived knowledge about COVID-19 risk factors, healthy behaviours (washing hands, use of masks), threat perceptions (perceived chances of contracting the virus) and emotional responses; and (2) to examine the role of gender as a factor moderating between perceived knowledge about the virus on the one hand and precautionary behaviours, perceived susceptibility and emotional responses on the other.

## Methods

### Procedure and participants

This study was a cross-sectional online survey conducted in Israel. The questionnaires were administered through the Qualtrics online platform (www.qualtrics.com). A total of 482 Israeli respondents aged 18–65 y visited the online survey during 12–21 March 2020. Participants (men=237; women=245) were recruited via social media (e.g. Facebook). To be included in the study, participants had to be aged >18 y and Hebrew speakers.

### Measures

*Precautionary behaviour* was measured by four items written by authors consistent with the precautionary guidelines issued by the Israeli Health Ministry. Scale validity was assessed by expert validity, a form of content validity. The scale was reviewed by a panel of four expert physicians. The aim of expert validity was to eliminate totally irrelevant items from the instrument and to rephrase or reword items related to the measured construct where necessary.^24^ Participants were asked to indicate how often they adopted various precautionary behaviours on a five-point scale ranging from ‘1=not at all’ to ‘5=very often’. A composite index of the average of all items was created, with a higher score indicating that participants exhibit more precautionary behaviours. Sample items include washing hands with soap and water or alcohol-based rub, facial covering, social/physical distancing (maintaining a 2 m distance between people in public spaces) and avoiding crowds, avoiding close contact with people with symptoms such as coughing or sneezing, refraining from shaking hands and covering mouth and nose when coughing or sneezing. The index exhibited strong internal consistency (Cronbach's α=0.76).

*Perceived knowledge about COVID-19 risk factors* was measured using a six-item COVID-19 risk factors knowledge^24^ test that assessed symptoms, diagnosis, risk factors, means of infection, ways to protect oneself from COVID-19 infection and knowledge about how to refer an individual suspected to have COVID-19 for further care. The validity of the scale was assessed by expert validity, as detailed in the above section on *precautionary behaviour*. Answers were rated on a five-point Likert-type scale ranging from ‘1=don't know anything’ to ‘5=know very much’. A composite index of the average of all items was created, with a higher score indicating higher levels of knowledge about COVID-19 risk factors. The index exhibited strong internal consistency (Cronbach's α=0.83).

*Emotional reactions to COVID-19* were assessed based on previous studies conducted among the lay public^25^ by means of three questions concerning stress, fear and worry deriving from COVID-19^24^ (e.g. ‘How much do you worry about COVID-19?’). Answers were rated on a five-point Likert-type scale, ranging from ‘1=not at all’ to ‘5=very much’. A composite index of the average of all items was created, with a higher score indicating higher levels of negative emotional reactions toward COVID-19. The index exhibited strong internal consistency (Cronbach's α=0.94).

*Perceived susceptibility* is a one-item measure used to assess participants’ evaluation of their likelihood of being infected by the virus.^24^ ‘How likely do you think it is that you will be infected by COVID-19?’ Answers were rated on a five-point Likert-type scale, ranging from ‘1=not at all likely’ to ‘5=very likely’.

*Sociodemographic variables* included gender, age, years of education, marital status (married/divorced/widow/single/other), number of children, profession, medical problems (yes/no), health status (bad/reasonable/good), home isolation since the COVID-19 outbreak (yes/no) and resources that can make it easier to cope with COVID-19 (more information regarding COVID-19/professional support/non-professional support/working from home/other).

### Statistical analyses

Data were analysed using SPSS version 25 (IBM, Armonk, NY, USA). Gender differences regarding the demographic and study variables were examined using χ^2^ tests, Z tests for independent proportions and *t*-tests for independent samples, in accordance with the scale of the variables. Pearson and point bi-serial correlations were calculated to assess the associations between the research variables. A multiple hierarchical regression was calculated to assess the contribution of the study variables to precautionary behaviour. The moderating role of gender was analysed with Hayes's PROCESS macro,^26^ model 1. All continuous variables were standardised. Bootstrapping was used, with 1000 resamples of the data and 95% bias-corrected confidence intervals. Confidence intervals that do not include zero mean effects were significant at the p=0.05 level.

## Results

Table 1 depicts the background details by group. The sample included 482 respondents, 50% men and 50% women. The participants’ mean age was 42.27 (range 18–65, SD=12.10) y, with no gender difference. Their average education was 16.19 (range 10–28, SD=3.22) y, with no gender difference. Most of the participants in both groups were married (about 69%), had about two children on average, had no health problems (about 87%) and reported being in good health (about 85%), with no gender difference.

**Table 1.** Participants’ characteristics by gender (N = 482)

Sociodemographic characteristics	Men (N = 237)	Women (N = 245)	
Mean age (SD), range (y)	42.20 (12.39), 18–65	42.34 (11.84), 18–65	t(475) = −0.12
Mean number of years of education (SD), range	16.24 (3.55), 10–28	16.13 (2.84), 10–28	t(417.94) = 0.35
Marital status (%)			Z = 0.49
Married	160 (67.5%)	167 (69.6%)	(married vs others)
Divorced	11 (4.6%)	15 (6.3%)	
Widow	0 (0.0%)	1 (0.4%)	
Single	58 (24.5%)	51 (21.3%)	
Other	8 (3.4%)	6 (2.5%)	
Mean number of children (SD), range	2.09 (1.43),0–7	2.01 (1.19),0–5	t(406.24) = 0.56
Health problems (%)			Z = 0.22
Yes	29 (12.2%)	31(12.9%)	
No	208 (87.8%)	209 (87.1%)	
Health status (%)			χ^2^(2) = 1.32
good	206 (86.9%)	202 (84.2%)	
fair	29 (12.2%)	37 (15.4%)	
poor	2 (0.8%)	1 (0.4%)	
Resources to make easier to cope with COVID-19 (%)			χ^2^(4) = 3.17
More information regarding COVID-19	52 (23.0%)	52 (22.7%)	
Professional support	36 (15.9%)	39 (17.0%)	
Non-professional support (friends and family)	17 (7.5%)	21 (9.2%)	
Working from home	75 (33.2%)	84 (36.7%)	
Other	46 (20.4%)	33 (14.4%)	

With respect to resources participants believed would help them cope with COVID-19, both men and women reported being interested in working from home (about 35%) and in receiving more information about the virus (about 23%), with no gender difference.

As depicted in Table 2, among both men and women, the mean levels of perceived susceptibility were moderate and perceived knowledge about COVID-19 risk factors was moderate-high, both with no gender difference. Women scored higher than men on both precautionary behaviour and emotional reactions.

**Table 2.** Independent t-tests for the study variables by gender (N = 482)

	Women (N = 245)		Men (N = 237)			
	M	SD	M	SD	*t*(df)	d
Precautionary behaviour	3.80	0.79	3.50	0.88	3.91 (475)***	0.36
Perceived knowledge about COVID-19	3.72	0.74	3.66	0.71	0.81 (475)	0.07
Perceived susceptibility	2.61	0.94	2.55	0.96	0.70 (409)	0.07
Emotional reactions	3.33	1.13	2.71	1.08	6.07 (475)***	0.56

***p < 0.001, range 1–5.

Moderate and significant positive Pearson correlations emerged between perceived knowledge about COVID-19 risk factors and precautionary behaviour, between emotional reactions and precautionary behaviour and between perceived susceptibility and emotional reactions. That is, greater perceived knowledge about COVID-19 risk factors was related to higher precautionary behaviour, higher emotional reactions were related to higher precautionary behaviour and greater perceived susceptibility was related to higher emotional reactions. Gender exhibited a negative relationship to precautionary behaviour and emotional reactions (point bi-serial correlations), such that women's scores were higher than men's (Table 3).

**Table 3.** Means, standard deviations, ranges and Pearson correlations for the study variables (N = 482)

Variables	1	2	3	4	5
1. Gender	-				
2. Precautionary behaviour	−0.18***	-			
3. Perceived knowledge about COVID-19 risk factors	−0.04	0.40***	-		
4. Perceived susceptibility	−0.04	0.08	0.07	-	
5. Emotional reactions	−0.27***	0.31***	0.01	0.30***	-
Mean		3.64	3.69	2.58	3.02
SD		0.85	0.73	0.95	1.15
Possible range		1–5	1–5	1–5	1–5
Actual range		1–5	1–5	1–5	1–5

***p < 0.001, for gender (1:men, 0:women), point bi-serial correlation.

A multiple regression was calculated for precautionary behaviour as the dependent variable, with gender (1:male, 0:female), perceived knowledge about COVID-19 risk factors, perceived susceptibility and emotional reactions as independent variables. Age exhibited a significant correlation with precautionary behaviour (*r*=0.25, p<0.001) and was thus controlled for. The results in Table 4 show that the regression model is significant, explaining 30% of the variance in precautionary behaviour. Precautionary behaviour was found to be higher among women and older participants, those with higher perceived knowledge about COVID-19 risk factors and those with higher emotional reactions.

**Table 4.** Multiple hierarchical regression for precautionary behaviour (N=482)

	B	SE	β	Adj. R^2^
Step 1				0.10***
Gender	−0.35	0.08	−0.20***	
Age	0.02	0.01	0.24***	
Step 2				0.30***
Gender	−0.17	0.07	−0.10*	
Age	0.01	0.01	0.21***	
Perceived knowledge about COVID-19	0.39	0.05	0.34***	
Perceived susceptibility	−0.04	0.04	−0.05	
Emotional reactions	0.25	0.03	0.34***	

*p < 0.05, **p < 0.01, ***p < 0.001. F(5, 405) = 36.08, p < 0.001.

The possibility that gender moderates the relationship between perceived knowledge about COVID-19 risk factors, perceived susceptibility, emotional reactions and precautionary behaviour was assessed with the Hayes^26^ process macro. That is, perceived knowledge about COVID-19 risk factors, perceived susceptibility and emotional reactions were defined as the independent variables, gender as the moderator and precautionary behaviour as the dependent variable. Age was controlled for.

**Figure 1.**
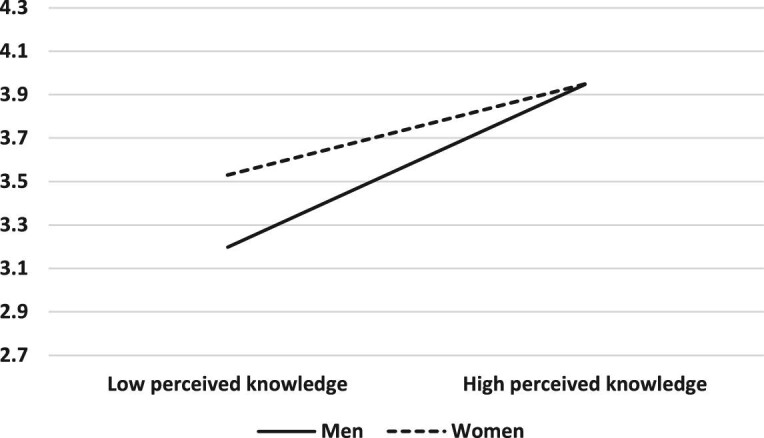
Gender as a moderating variable between perceived knowledge and precautionary behavior.

The moderating role of gender was found to be significant in the relationship between perceived knowledge about COVID-19 risk factors and precautionary behaviour (B=0.16, SE=0.07, β=0.13, p=0.02, 95% CI 0.03 to 0.30). When men and women perceived that their knowledge about COVID-19 risk factors was high, they exhibited similar precautionary behaviours. By contrast, when men and women perceived that their knowledge about COVID-19 risk factors was low, women showed more precautionary behaviours than men (Figure 1). Gender was not found to moderate the relationship between either perceived susceptibility or emotional reactions and precautionary behaviour.

## Discussion

This study examined gender differences in perceived knowledge of COVID-19 risk factors, perceived susceptibility, emotional responses and health behaviour among 482 men and women from the general public in Israel. A significant gender difference emerged for precautionary behaviour and emotional responses, with women reporting higher levels on each of these measures. Nevertheless, no gender differences emerged in extent of perceived susceptibility or extent of perceived knowledge of COVID-19 risk factors. The moderating role of gender was found to be significant in the relationship between perceived knowledge of COVID-19 risk factors and precautionary behaviour.

This study found that women report higher levels of preventive behaviour than men. This is in line with previous studies in which women reported higher levels of preventive behaviour regarding COVID-19 than men.^27–29^ In a study in the UK among 2459 participants, men expressed lower intentions than women to wear a face covering, a finding that can be partly explained by the fact that men believe more strongly than women that they will be relatively unaffected by the disease.^30^ These gender differences may suggest that women believe that by being more careful and concerned they can reduce the risk of being severely affected by COVID-19.^31^ In the current study, women report higher levels of emotional reactions to COVID-19.

Gender has been found to be one of the strongest and most consistent predictors of fear of crime and trauma. Women are more afraid of crime than men, although men are more likely than women to be the victim of a crime.^32,33^ One possible explanation for the findings of this study regarding the higher levels of health behaviours and emotional responses among women can be found in the context of other virus outbreaks. During the Ebola outbreak, women experienced changes in their traditional roles at home and at work, along with uncertainty about their lives and their families. These feelings were particularly high among pregnant women.^34^ Evidence from the Zika virus outbreak points to deleterious effects on women's well-being, primarily due to problems in gaining access to health services.^12^ In a study among parents of children with the Zika virus, gender predicted parents’ mental health, with mothers tending to present higher levels of depression and anxiety than fathers.^35^ A recent study among the Chinese public^36^ suggests that perceived severity of COVID-19 may have a negative main effect on mental health outcomes.

Another possible explanation for the high levels of emotional responses among women is related to the nature of COVID-19. Part of the necessary response to the COVID-19 outbreak entails limiting social contact, particularly with those who exhibit symptoms or are at increased risk of contracting the virus. While self-isolation can help contain and control the spread of infectious diseases, isolation also has major negative psychological effects.^37^ Women may have more trouble with isolation than men and may need more support.^18^ They may also express more anxiety and concern about their children and their family, leading them to adopt more healthy behaviours.^11^

It should be noted that the data for this study were collected in March 2020, when the epidemic first broke out in Israel. This timing may explain the high level of emotional distress. It is important to examine emotional responses at other time points to determine whether people became accustomed to the situation or whether their emotional responses became exacerbated.

The current study found no gender differences regarding perceived knowledge about COVID-19 risk factors. This finding is supported by studies in Malaysia and Pakistan among public or healthcare workers that found no gender differences regarding perceived knowledge about COVID-19 risk factors.^38,39^ The same is true in previous studies, for example, those focusing on Ebola, AIDS and pandemic influenza.^40–43^ Studies in Singapore and in Beijing that examined knowledge about the SARS virus also did not find any differences between men and women.^44,45^ The fact that the current study found no gender differences regarding perceived knowledge about COVID-19 risk factors may be attributed to the intensive public health education campaign in Israel. Note that the participants of this study had a high average level of education. Thus, level of perceived knowledge may have differed among other socioeconomic groups. This topic should be examined in different population groups.

Although no gender differences regarding knowledge emerged, the moderating role of gender was found significant in the relationship between perceived knowledge and precautionary behaviour. Human behaviour and knowledge assessment during the crisis are critical in the overall efforts to contain the outbreak. Timely and large-scale dissemination of information about how to prevent and control COVID-19 aimed at the entire public through various mass media is important to keep residents well informed of preventive measures and progress in controlling the infection, thus empowering their ability to adopt health behaviour measures.

The present results are cross-sectional and thus cannot explain the causes either of knowledge or of behaviour. Nevertheless, they do suggest that even although women and men were equally exposed to information and guidelines, women may have been more responsive to the guidelines and were more motivated to act and comply. The behaviour of the general public is important to keep the spread of this deadly virus in check in developing countries.^6,46^

### Limitations

This study has a number of limitations. Its main limitation is its cross-sectional design. As a result, conclusions about directionality or causality in the relationships should be treated with caution. A second limitation is that the present study is based on participants’ self-report questionnaires. Because the data were collected from social networks, we cannot assess the rate of response. Nevertheless, the online recruitment method helped us collect data from a diverse sample within a short time, given the restrictions on physical mobility due to the lockdown. People responded online and the mean number of years of education was relatively high. As the virus spreads across the globe, the current findings have the potential to be useful for further research.

In conclusion, responses to COVID-19 are marked by gender differences, with women reporting higher levels of precautionary behaviour and emotional reactions to COVID-19 than men. No gender differences emerged in perceived knowledge about COVID-19 risk factors. These findings are important in understanding the behaviour of the general public in coping with COVID-19 as well as in understanding gender differences in public health. We recommend extending this investigation through data sampling at different points in time as the virus spreads to other countries.

## Data Availability

The authors have the research data, which are available upon request.

## Availability of data and materials

The authors have the research data, which are available upon request.

## Patient and public involvement

It was not appropriate or possible to involve patients or the public in the design, conduct, reporting or dissemination plans of our research.

## References

[1] Chan JF , Yuan S, Kok KH, et al. A familial cluster of pneumonia associated with the 2019 novel coronavirus indicating person-to-person transmission: a study of a family cluster. Lancet. 2020;395(10223):514–23. 31986261 10.1016/S0140-6736(20)30154-9 PMC7159286

[2] Huang C , Wang Y, Li X, et al. Clinical features of patients infected with 2019 novel coronavirus in Wuhan, China. Lancet. 2020;395(10223):497–506. 31986264 10.1016/S0140-6736(20)30183-5 PMC7159299

[3] Li H , Chen X, Huang H. The novel coronavirus outbreak: what can be learned from China in public reporting? Global Health Res Policy. 2020;5(1):1–3. 10.1186/s41256-020-00140-9 PMC7061470 32166128

[4] Eurosurveillance Editorial Team . Note from the editors: novel coronavirus (2019-nCoV). Euro Surveill. 2020;25(3):2001231. 10.2807/1560-7917.ES.2020.25.3.2001231 PMC6988271 31992390

[5] Corley MJ , Ndhlovu LC. DNA methylation analysis of the COVID-19 host cell receptor, Angiotensin I Converting Enzyme 2 Gene (ACE2) in the respiratory system reveal age and gender differences. Preprints. 2020;2020030295. Available at https://www.preprints.org/manuscript/202003.0295/v1 [accessed October 10, 2020].

[6] Levkovich I , Shinan-Altman S. Impact of the COVID-19 pandemic on stress and emotional reactions in Israel: a mixed-methods study. Int Health. 2020, ihaa081. 10.1093/inthealth/ihaa081. PMC7665529 33049782

[7] Novel Coronavirus Pneumonia Emergency Response Epidemiology Team . The epidemiological characteristics of an outbreak of 2019 novel coronavirus diseases (COVID-19) in China. Chin J Epidemiol. 2020;4:145–51.

[8] Jiang F , Deng L, Zhang L, et al. Review of the clinical characteristics of coronavirus disease 2019 (COVID-19). J Gen Intern Med. 2020;35(5):1545–9. 32133578 10.1007/s11606-020-05762-w PMC7088708

[9] Chen N , Zhou M, Dong X, et al. Epidemiological and clinical characteristics of 99 cases of 2019 novel coronavirus pneumonia in Wuhan, China: a descriptive study. Lancet. 2020;395(10223):507–13. 32007143 10.1016/S0140-6736(20)30211-7 PMC7135076

[10] Liu S , Zhang M, Yang L, et al. Prevalence and patterns of tobacco smoking among Chinese adult men and women: findings of the 2010 national smoking survey. J Epidemiol Community Health. 2017;71(2):154–61. 27660401 10.1136/jech-2016-207805 PMC5284482

[11] Lau JTF , Yang X, Tsui HY, et al. SARS related preventive and risk behaviours practised by Hong Kong-mainland China cross border travellers during the outbreak of the SARS epidemic in Hong Kong. J Epidemiol Community Health. 2004;58(12):988–96. 15547057 10.1136/jech.2003.017483 PMC1732647

[12] Davies SE , Bennett B. A gendered human rights analysis of Ebola and Zika: Locating gender in global health emergencies. J Int Aff. 2016;92(5):1041–60.

[13] Plourde AR , Bloch EM. A literature review of Zika virus. Emerg Infect Dis. 2016;22(7):1185. 27070380 10.3201/eid2207.151990 PMC4918175

[14] Díaz A , Soriano JF, Beleña Á. Perceived Vulnerability to Disease Questionnaire: Factor structure, psychometric properties and gender differences. Pers Ind Diff. 2016;101:42–9.

[15] Faulkner J , Schaller M, Park JH, et al. Evolved disease-avoidance mechanisms and contemporary xenophobic attitudes. Group Process Intergr Relat 2004;7:333–53.

[16] Díaz A , Beleña Á, Zueco J. The role of age and gender in perceived vulnerability to infectious diseases. Int J Environ Res Public Health. 2020;17(2):485. 10.3390/ijerph17020485 PMC7014162 31940870

[17] Hill SE , Prokosch ML, DelPriore DJ. The impact of perceived disease threat on women's desire for novel dating and sexual partners: Is variety the best medicine? J Pers SocPsychol. 2015;109(2):244. 10.1037/pspi0000024 26030057

[18] Liu N , Zhang F, Wei C, et al. Prevalence and predictors of PTSS during COVID-19 outbreak in China hardest-hit areas: Gender differences matter. Psychiatry Res. 2020;16:112921. 10.1016/j.psychres.2020.112921 PMC7102622 32240896

[19] Kaljee L , Zhang L, Langhaug L, et al. A randomized-control trial for the teachers’ diploma programme on psychosocial care, support and protection in Zambian government primary schools. Psychol Health Med. 2017;22:381–92. 26965476 10.1080/13548506.2016.1153682

[20] Senst L , Baimoukhametova D, Sterley TL, et al. Sexually dimorphic neuronal responses to social isolation. Elife. 2016;5:e18726. 27725087 10.7554/eLife.18726 PMC5059136

[21] Spagnolo PA , Manson JE, Joffe H. Sex and gender differences in health: What the COVID-19 pandemic can teach us. Ann Intern Med. 2020;173:385–6. 32384135 10.7326/M20-1941 PMC7249504

[22] Thelwall M , Thelwall S. Covid-19 tweeting in English: Gender differences. EPI. 2020;29:3.

[23] Gao WB , Chen ZY, Wang YN. The dynamic analysis of public concern during SARS epidemic period. Chin Ment Health J. 2003;17:594–6.

[24] Shinan-Altman S , Levkovich I, Tavori G. Healthcare utilization among breast cancer patients during the COVID-19 outbreak. Palliat Support Care. 2020;18(4):385–91. 32594966 10.1017/S1478951520000516 PMC7360944

[25] Werner P , Goldberg S, Mandel S, et al. Gender differences in lay persons’ beliefs and knowledge about Alzheimer's disease (AD): A national representative study of Israeli adults. Arch Gerontol Geriatr. 2013;56(2):400–4. 23219063 10.1016/j.archger.2012.11.001

[26] Hayes AF. Introduction to Mediation, Moderation, and Conditional Process Analysis: A Regression-Based Approach. New York: Guilford Publications; 2017.

[27] Yıldırım M , Güler A. COVID-19 severity, self-efficacy, knowledge, preventive behaviors, and mental health in Turkey. Death Stud. 2020;1–8. doi:10.1080/07481187.2020.1793434. 10.1080/07481187.2020.1793434 32673183

[28] Ahorsu DK , Imani V, Lin CY, et al. Associations between fear of COVID-19, mental health, and preventive behaviours across pregnant women and husbands: An actor-partner interdependence modelling. Int J Ment Health Addict. 2020;11:1–15. 10.1007/s11469-020-00340-x PMC7289236 32837427

[29] Yıldırım M , Geçer E, Akgül Ö. The impacts of vulnerability, perceived risk, and fear on preventive behaviours against COVID-19. Psychol Health Med. 2020;26(1):1–9. 32490689 10.1080/13548506.2020.1776891

[30] Capraro V , Barcelo H. The effect of messaging and gender on intentions to wear a face covering to slow down COVID-19 transmission. arXiv. 2020. Available at https://arxiv.org/abs/2005.05467 [accessed October 9, 2020]. 10.1002/acp.3793 PMC8013666 33821089

[31] Galasso V , Pons V, Profeta P, et al. Gender differences in COVID-19 related attitudes and behavior: Evidence from a panel survey in eight OECD countries (No. w27359). NBER Work Pap Ser. 2020. 10.1073/pnas.2012520117 PMC7959517 33060298

[32] May DC , Herbert J, Cline K, et al. Predictors of fear and risk of terrorism in a rural state. Int J Rural Criminol. 2011;1:1–22.

[33] Mansoor T , Hasan R. Gender differences in the fear of crime victimization and precautionary behaviours. Pakistan J Gend Stud. 2016:165–94.

[34] Nkangu MN , Olatunde OA, Yaya S. The perspective of gender on the Ebola virus using a risk management and population health framework: a scoping review. Infect Dis Poverty. 2017;6:135. 29017587 10.1186/s40249-017-0346-7 PMC5635524

[35] de Souza LE , de Lima TJ, Ribeiro EM, et al. Mental health of parents of children with congenital Zika virus syndrome in Brazil. J Child Fam Stud. 2018;27:1207–15.

[36] Li JB , Yang A, Dou K, et al. Self-control moderates the association between perceived severity of the coronavirus disease 2019 (COVID-19) and mental health problems among the Chinese public. PsyArXiv. 2020. Available at https://psyarxiv.com/2xadq/ [accessed October 9, 2020]. 10.3390/ijerph17134820 PMC7370094 32635495

[37] Day T , Park A, Madras N, et al. When is quarantine a useful control strategy for emerging infectious diseases? Am J Epidemiol. 2006;163:479–85. 16421244 10.1093/aje/kwj056 PMC7109638

[38] Mohd Hanafiah K , Wan CD. Public knowledge, perception and communication behavior surrounding COVID-19 in Malaysia. Advance Preprint. Available at https://advance.sagepub.com/articles/Public_knowledge_perception_and_communication_behavior_surrounding_COVID-19_in_Malaysia/12102816 [accessed October 9, 2020].

[39] Saqlain M , Munir MM, Rehman SU, et al. Knowledge, attitude, practice and perceived barriers among healthcare workers regarding COVID-19: a cross-sectional survey from Pakistan. J Hosp Infect. 2020;105:419–23. 32437822 10.1016/j.jhin.2020.05.007 PMC7211584

[40] Kamate SK , Agrawal A, Chaudhary H, et al. Public knowledge, attitude and behavioural changes in an Indian population during the Influenza A (H1N1) outbreak. J Infect Dev Countr. 2010;4:7–14. 10.3855/jidc.501 20130372

[41] Farid-ul-Hasnain S , Johansson E, Krantz G. What do young adults know about the HIV/AIDS epidemic? Findings from a population based study in Karachi, Pakistan. BMC Infect Dis. 2009;9:38. 19323807 10.1186/1471-2334-9-38 PMC2678138

[42] Gupta N , Mehta N, Gupta P, et al. Knowledge regarding Ebola Hemorrhagic Fever among private dental practitioners in Tricity, India: A cross-sectional questionnaire study. Niger J Med. 2015;56:138. 10.4103/0300-1652.153405 PMC4382605 25838631

[43] Akan H , Gurol Y, Izbirak G, et al. Knowledge and attitudes of university students toward pandemic influenza: a cross-sectional study from Turkey. BMC Public Health. 2010;10:413. 20626872 10.1186/1471-2458-10-413 PMC2918554

[44] Deurenberg-Yap M , Foo LL, Low YY, et al. The Singaporean response to the SARS outbreak: knowledge sufficiency versus public trust. Health Promot Int. 2005;20:320–6. 15964886 10.1093/heapro/dai010 PMC7108623

[45] Yang X , Yao C, Wang K, et al. Severe acute respiratory syndrome: knowledge, belief and behavior among Beijing residents. Asia Pac Fam Med. 2006;5:1–6.

[46] ul Haq S , Shahbaz P, Boz I. Knowledge, behavior and precautionary measures related to COVID-19 pandemic among the general public of Punjab province, Pakistan. J Infect Dev Ctries. 2020;14(8):823–35. 32903224 10.3855/jidc.12851

